# Supplementary material for: Transcriptome-Based Revelation of the Effects of Sleep Deprivation on Hepatic Metabolic Rhythms in Tibetan Sheep (Ovis aries)
Source: Animals (Basel). 2024 Nov 5;14(22):3165. doi: 10.3390/ani14223165 (PMC11591132; doi:10.3390/ani14223165)
Supplement: Supplementary file 1 [file animals-14-03165-s001.zip › animals-3261031-supplementary.pdf]

Supplementary Materials

Janakhire-Tarpstra-Kendall-Cycle

Janakhire-Tarpstra-Kendall-Cycle (JTK CYCLE) is a non-parametric algorithm. Its purpose is to identify rhythmic components in large, genome-scale data sets and estimate their period length, phase, and amplitude. Circadian rhythms are oscillations of physiology, behavior, and metabolism that have period lengths near 24 hours. In several model organisms and humans, circadian clock genes have been characterized and found to be transcription factors. Because of this, researchers have used microarrays to characterize global regulation of gene expression and algorithmic approaches to detect cycling. This article presents a new algorithm, JTK CYCLE, designed to efficiently identify and characterize cycling variables in large data sets. Compared with COSOPT and the Fisher's G test, two commonly used methods for detecting cycling transcripts, JTK_CYCLE distinguishes between rhythmic and nonrhythmic transcripts more reliably and efficiently. JTK CYCLE's increased resistance to outliers results in considerably greater sensitivity and specificity. Moreover, JTK CYCLE accurately measures the period, phase, and amplitude of cycling transcripts, facilitating downstream analyses. Finally, JTK CYCLE is several orders of magnitude faster than COSOPT, making it ideal for large-scale data sets[43].

Supplementary Figures

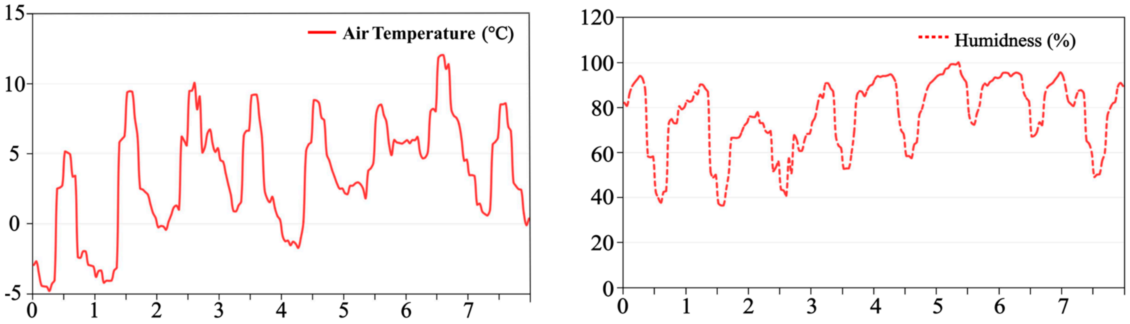

FIGURE S1 | The average temperature(left) and average relative humidity(right) were recorded throughout the duration of the experiment.

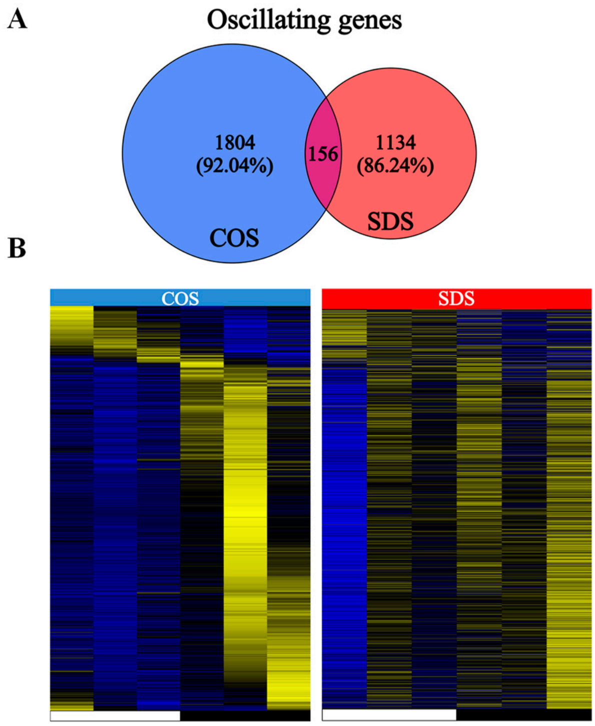

FIGURE S2| Sleep deprivation disrupted the circadian rhythm of liver genes in Tibetan sheep. (A) Vene diagram displaying the number (top) and ratio (bottom) of oscillating genes in liver samples isolated from Tibetan sheep in the COS and SDS groups (ADJ.P < 0.01). (B) Heat map showing oscillating genes in the COS (left) or SD (right) groups ($p < 0.05$). (C) KEGG enriched pathways for the expression of COS group oscillating genes and SDS group oscillating genes ($p < 0.05$).

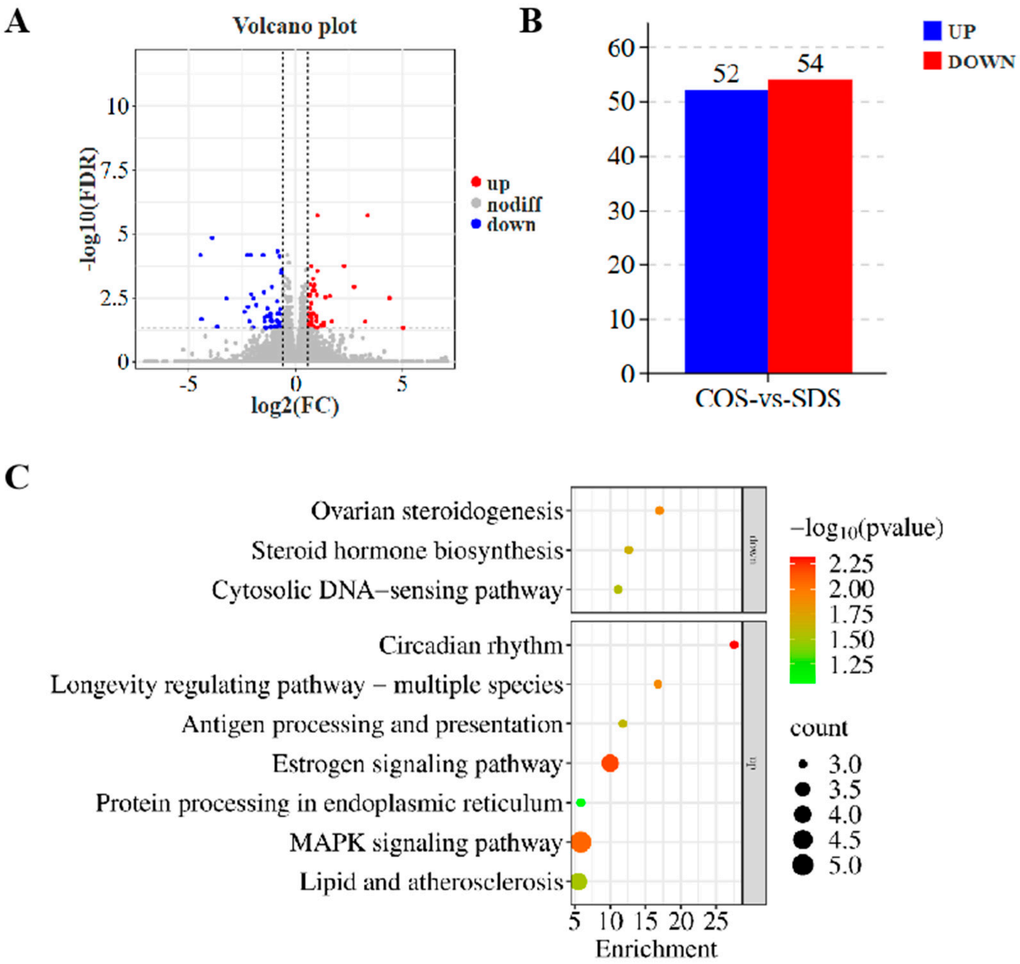

FIGURE S3| Hepatic transcriptomic analysis of livers from SD treated Tibetan sheep. (A-B) Volcano plot and bar graph based on the changed genes of the SD group compared with the COS group (FC: >log2(1.5) or <log2(-1.5); FDR < 0.05). (C) KEGG enriched pathways for the expression of COS group DEG$_S$ and SDS group DEG$_S$ ($p < 0.05$).

Supplementary Tables

Table S1. Composition of the basal diets.

Ingredients (%)	Content	Nutrient composition (%)	Content
Corn	32.5	Crude protein	13.350
Soybean meal	12	Metabolizable energy[2](MJ/kg)	13.005
Wheat bran	2.5	Neutral detergent fiber	40.155
Straw	22.5	Acid detergent fiber	27.775
Sweet sorghum silage	27.5		
Sodium chloride	0.25		
Sodium bicarbonate	0.75		
Premix[1]	2		
Total	100		

[1] Formulated to provide (per kilogram of premix) 50 KIU of Vitamin A, 10 KIU of vitamin D3, 450 mg of Vitamin E, 2100 mg of Zn, 8 mg of Se, 900 mg of I, 2100 mg of Fe, 10 mg of Co, 30 mg of Mn, and 1 350 mg of Cu;

[2] Calculated according to Ministry of Agriculture of P.R. China, 2004.

Table S2. Summary of data quality and genome alignment

Sample	Clean Reads	Clean Base(bp)	GC (%)	Q30 (%)	Total Mapped(%)	Unique Mapped(%)
CS_ZT2_1	44948970	6721783748	49.1	94.71	97.93	92.49
CS_ZT2_2	42434076	6339570410	48.94	94.51	97.72	92.71
CS_ZT2_3	38834280	5795923080	49.38	94.84	97.65	92.56
CS_ZT2_4	42679184	6379023036	49.24	94.91	97.76	92.28
CS_ZT6_1	36149616	5399927497	49.16	94.46	97.5	92.42
CS_ZT6_2	39267578	5864617336	48.57	94.85	97.72	92.77
CS_ZT6_3	39382208	5879536963	48.68	94.27	97.88	92.94
CS_ZT6_4	43324350	6477853573	48.8	94.47	97.62	92.43
CS_ZT10_1	40092622	5989215494	48.63	94.29	97.6	92.57
CS_ZT10_2	41469658	6194073417	48.97	94.77	97.89	92.91
CS_ZT10_3	39905028	5956555178	48.43	94.99	97.93	93
CS_ZT10_4	38702656	5775392605	48.23	94.21	97.69	92.61
CS_ZT14_1	39026282	5832007088	48.48	94.53	97.62	92.51
CS_ZT14_2	35961980	5369094255	48.79	94.87	97.65	92.69
CS_ZT14_3	43835808	6547483145	48.19	94.56	97.49	92.44
CS_ZT14_4	38221610	5707614941	48.53	94.75	97.68	92.43
CS_ZT18_1	45135936	6745397106	47.28	94.53	97.26	91.81
CS_ZT18_2	41806964	6249413524	46.52	94.89	97.62	92.32
CS_ZT18_3	40256602	6014660409	49.19	94.63	97.56	92.58
CS_ZT18_4	41346498	6172254510	47.47	95.18	97.88	92.46
CS_ZT22_1	42975734	6416998229	49.11	94.66	97.13	92.36
CS_ZT22_2	38619614	5765844371	48.09	94.66	97.51	92.52
CS_ZT22_3	43556576	6499873782	48.82	94.9	97.66	92.6
CS_ZT22_4	38093708	5675873486	48.71	94.69	97.59	92.48
SDS_ZT2_1	40119970	5996720240	49.09	95.09	97.8	92.72
SDS_ZT2_2	41674748	6222474302	48.98	94.92	97.74	92.95
SDS_ZT2_3	37753772	5636148996	48.84	94.98	97.78	92.5
SDS_ZT2_4	41825448	6249037752	49.14	95.1	97.72	92.57
SDS_ZT6_1	43157348	6448100872	48.86	94.94	97.72	92.59
SDS_ZT6_2	41022108	6121982961	48.75	95.24	97.69	92.46
SDS_ZT6_3	42056038	6278461750	48.87	94.78	97.68	92.54
SDS_ZT6_4	40962186	6122216994	48.76	94.98	97.63	92.59
SDS_ZT10_1	41480594	6194181758	48.78	95.03	97.79	92.31
SDS_ZT10_2	42380686	6328991746	48.67	94.62	97.66	92.74
SDS_ZT10_3	42861488	6408203181	48.94	94.09	97.55	92.3
SDS_ZT10_4	42026646	6283231524	48.85	94.58	97.85	92.63
SDS_ZT14_1	42040080	6274530731	49.11	94.9	97.68	92.14
SDS_ZT14_2	38914324	5808741189	48.97	94.56	97.72	92.46
SDS_ZT14_3	41764716	6229791501	48.99	94.72	97.59	92.49
SDS_ZT14_4	42177406	6298709357	48.87	94.89	97.75	92.47
SDS_ZT18_1	36479272	5449123122	49.22	95.09	97.88	92.88

SDS_ZT18_2	36784708	5486730810	49.01	95.03	97.89	92.46
SDS_ZT18_3	37853528	5653829675	48.05	94.77	97.86	92.39
SDS_ZT18_4	39701776	5927939524	48.44	95.05	97.97	92.7
SDS_ZT22_1	42361138	6328716576	48.96	95.04	97.83	92.81
SDS_ZT22_2	42030664	6278043621	49.26	94.82	97.54	92.25
SDS_ZT22_3	36148128	5399225162	48.73	94.81	97.83	92.92
SDS_ZT22_4	39015032	5825737731	48.95	94.72	97.72%	92.51

Table S3. Details of DEGs screened from the COS vs SDS

Symbol	log2(fc)	FDR	id	Regulated Type
LOC101111915	-1.72	9.56E-13	ncbi_101111915	down
RHOB	1.06	1.91E-06	ncbi_100158235	up
IL32	3.40	1.91E-06	ncbi_114110569	up
LOC101108414	-3.88	1.50E-05	ncbi_101108414	down
LOC105614315	-0.82	4.99E-05	ncbi_105614315	down
LOC105602066	-4.42	6.95E-05	ncbi_105602066	down
TFF2	-2.23	6.95E-05	ncbi_101104197	down
HBB	-2.09	6.95E-05	ncbi_100049064	down
H1-6	-1.50	6.95E-05	ncbi_101112356	down
LOC101112606	-0.73	7.80E-05	ncbi_101112606	down
BHLHE40	0.77	1.87E-04	ncbi_100169939	up
RGS16	2.30	1.87E-04	ncbi_101103743	up
CAMK2N1	-0.64	2.66E-04	ncbi_101102884	down
LOC121815981	1.06	2.89E-04	ncbi_121815981	up
ENDOG	-0.64	3.38E-04	ncbi_780458	down
TNFRSF21	0.83	5.83E-04	ncbi_101117284	up
C14H19orf12	0.71	9.55E-04	ncbi_101106870	up
MTHFD1L	0.92	1.01E-03	ncbi_101102245	up
SLC20A1	0.87	1.16E-03	ncbi_101112466	up
LOC114116834	-1.09	1.21E-03	ncbi_114116834	down
NCAM1	2.76	1.21E-03	ncbi_101112759	up
HSPA1A	0.81	1.66E-03	ncbi_100913152	up
--	0.93	1.67E-03	MSTRG.1971	up
CENPW	-1.46	1.96E-03	ncbi_101117252	down
LOC114110673	-2.05	2.34E-03	ncbi_114110673	down
LPCAT3	0.71	2.43E-03	ncbi_101108119	up
LOC101104661	1.03	2.47E-03	ncbi_101104661	up
B3GALT6	0.63	2.52E-03	ncbi_101105768	up
BAG3	0.68	2.58E-03	ncbi_101108530	up
ADM2	1.64	2.72E-03	ncbi_106991013	up
GPX3	1.44	3.08E-03	ncbi_101104397	up
MROH7	4.42	3.28E-03	ncbi_101122145	up
FOSB	-1.95	3.38E-03	ncbi_101117101	down
LOC101109377	-3.21	3.40E-03	ncbi_101109377	down
HMGB3	-0.84	4.42E-03	ncbi_101107173	down
NFIL3	0.76	5.18E-03	ncbi_100217409	up
LOC101108797	-1.82	6.19E-03	ncbi_101108797	down
UGGT2	-2.21	7.31E-03	ncbi_101108761	down
CXCL10	-1.29	8.28E-03	ncbi_442997	down
TIMM10B	0.68	8.28E-03	ncbi_101117359	up
INSIG1	0.70	8.28E-03	ncbi_101106422	up

IDI1	-0.70	8.86E-03	ncbi_101108851	down
ALAS2	-2.36	1.13E-02	ncbi_101115684	down
ZBP1	-0.76	1.31E-02	ncbi_101104169	down
LOC101121034	0.85	1.31E-02	ncbi_101121034	up
ARMC12	-1.16	1.33E-02	ncbi_101118823	down
LOC101110974	-0.84	1.33E-02	ncbi_101110974	down
HSD17B1	-0.75	1.33E-02	ncbi_101110778	down
PFKFB3	0.62	1.33E-02	ncbi_101107621	up
NABP1	0.64	1.33E-02	ncbi_101118080	up
CREB3L3	0.73	1.35E-02	ncbi_101113586	up
PDZK1IP1	0.64	1.41E-02	ncbi_101106377	up
SAT2	-0.61	1.55E-02	ncbi_101103248	down
LOC105601949	-1.34	1.67E-02	ncbi_105601949	down
HSPA6	1.00	1.67E-02	ncbi_101107064	up
SGK1	0.67	1.68E-02	ncbi_101107709	up
POMC	-1.16	1.77E-02	ncbi_443212	down
LOC105602080	0.71	1.80E-02	ncbi_105602080	up
LOC105603225	-1.43	1.90E-02	ncbi_105603225	down
FAM107A	0.79	2.02E-02	ncbi_101121206	up
--	-4.37	2.17E-02	MSTRG.11411	down
CHP2	-1.14	2.49E-02	ncbi_101118159	down
METRN	-0.86	2.51E-02	ncbi_101110897	down
FGF21	1.01	2.52E-02	ncbi_101108510	up
--	-1.07	2.58E-02	MSTRG.6010	down
--	-1.43	2.62E-02	MSTRG.6773	down
LOC101102466	-2.13	2.62E-02	ncbi_101102466	down
RIGI	-0.82	2.62E-02	ncbi_101107257	down
INHBA	0.77	2.62E-02	ncbi_443524	up
DKK1	0.79	2.62E-02	ncbi_101116876	up
CTLA4	1.72	2.66E-02	ncbi_443040	up
MMP12	3.28	2.71E-02	ncbi_101119157	up
GADD45A	0.61	2.86E-02	ncbi_101115240	up
IFI44	-0.61	2.99E-02	ncbi_101110473	down
NNAT	1.35	3.00E-02	ncbi_101122618	up
CYSLTR2	-0.70	3.01E-02	ncbi_101114623	down
--	0.78	3.20E-02	MSTRG.20213	up
CDC25A	-0.69	3.43E-02	ncbi_101113178	down
ZBED5	-0.65	3.43E-02	ncbi_101122975	down
PER1	0.64	3.45E-02	ncbi_100174984	up
DLEC1	0.65	3.45E-02	ncbi_101115235	up
PROSER2	-0.73	3.57E-02	ncbi_101116497	down
SLC7A1	0.67	3.60E-02	ncbi_443452	up
CNTNAP2	-0.59	3.78E-02	ncbi_101106322	down
USP2	1.23	3.80E-02	ncbi_101109478	up

--	1.35	3.80E-02	MSTRG.14366	up
WNT5A	-0.77	3.89E-02	ncbi_101109745	down
PPFIA1	0.86	4.15E-02	MSTRG.21711	up
ID1	0.78	4.16E-02	ncbi_101111650	up
LOC121816006	-3.64	4.26E-02	ncbi_121816006	down
--	-1.13	4.26E-02	MSTRG.10916	down
NKD1	-0.95	4.26E-02	ncbi_101116855	down
ITGBL1	-1.97	4.50E-02	ncbi_101112990	down
NPTX2	-1.40	4.50E-02	ncbi_101104118	down
FCER1A	-0.84	4.50E-02	ncbi_100144427	down
ZC3H12A	0.63	4.50E-02	ncbi_101118325	up
--	-0.59	4.55E-02	MSTRG.22836	down
PRSS33	-0.94	4.57E-02	ncbi_105604615	down
--	-1.14	4.62E-02	MSTRG.13751	down
LOC105616911	0.73	4.62E-02	ncbi_105616911	up
--	1.06	4.62E-02	MSTRG.17578	up
PLEKHG6	-0.63	4.71E-02	ncbi_101110410	down
LOC114117018	5.05	4.80E-02	ncbi_114117018	up
TAF4B	0.79	4.81E-02	ncbi_101117382	up
ABCA13	-1.38	4.88E-02	ncbi_121819380	down
ERVK-7	0.89	4.98E-02	MSTRG.22388	up

Table S4. The sequence of primers in RT-qPCR

Gene	Primer(5' to 3')
GAPDH	GTCAAGGCAGAGAACGGGAA
	TCACAAACATGGGAGCGTCA
LIPG	CATTCAGATGCCTGTGGGTCA
	TTGAAGCGGTTGGAGTCTGTG
TNFSF10	AGTTCTGCGTGCTGATCCTC
	GTCCCAGGAACGGTCATCTT
ACSL3	GCGGCACATCATCACTGTAGA
	TTGCTGAGGGGTTTGTTTTCC
LPCAT3	CTGTTGGCTGGTCACGGAAG
	GGCGTTGGTGTTGATGTTGAA
ENDOG	GCGTCGTACGTGCTGTGTTA
	GTGGTACGCATGCACCGA
